# An updated systematic review, meta-analysis, and trial sequential analysis of the efficacy and safety of en bloc transurethral resection vs. conventional transurethral resection for nonmuscle-invasive bladder tumor

**DOI:** 10.1097/JS9.0000000000002291

**Published:** 2025-01-10

**Authors:** Zhunan Xu, Qihua Wang, Bing Li, Xuexue Hao, Congzhe Ren, Xiangyu Chen, Li Liu, Xiaoqiang Liu

**Affiliations:** Department of Urology, Tianjin Medical University General Hospital, Tianjin, China

**Keywords:** early oral feeding, gastrectomy, gastric cancer, meta-analysis, traditional oral feeding

## Abstract

**Objective:**

En bloc transurethral resection of bladder tumor (ERBT) for nonmuscular-invasive bladder tumor (NMIBC) has been used in clinical practice, but its efficacy and safety have not been conclusional. We aimed to evaluate the efficacy and safety of ERBT and conventional transurethral resection of bladder tumor (cTURBT) for NMIBC.

**Methods:**

Randomized controlled trials (RCTs) comparing ERBT and cTURBT in the treatment of NMIBC were searched in Pubmed, Embase, Clinicaltrials.gov, and Cochrane; 12 RCTs were included for systematic review and meta-analysis using RevMan 5.4.

**Results:**

A total of 12 RCTs involving 2097 patients with NMIBC were included and analyzed. The results showed the rate of identification of detrusor muscle in specimens [odds ratio (OR) 1.90; *P* = 0.03] was higher in ERBT group, and bladder perforation (OR 0.30; *P* = 0.004), obturator nerve reflex (OR 0.18; *P* = 0.001), catheter indwelling time (MD −0.64; *P* = 0.002), length of hospital stay (MD −0.58; *P* = 0.002), tumor recurrence rate 3 months after surgery (OR 0.42; *P* = 0.03), tumor recurrence rate 6 months after surgery (OR 0.21; *P* = 0.007), the recurrence rate of the same site 1 year after surgery (OR 0.23; *P* < 0.0001) and bleeding rate (OR 0.30; *P* = 0.0005) were significantly lower in ERBT group than that in cTURBT group. There was no significant difference in residual tumor (OR 0.62; *P* = 0.07), Re-TURBT (OR 0.71; *P* = 0.19), hemoglobin deficit (MD −0.81; *P* = 0.29), urethral stricture (OR 0.67; P = 0.42), resection time (MD 2.31; *P* = 0.16), operative time (MD 1.17; *P* = 0.49), 1 year (OR 0.61; *P* = 0.13), 2 years (OR 0.94; *P* = 0.76), 3-year tumor recurrence rate (OR 1.03; *P* = 0.86) and the risk of progression at 12 month (OR 0.68; *P* = 0.79) between the two groups.

**Conclusions:**

Our results showed that ERBT can improve the rate of identification of detrusor muscle in specimens and reduce the 3-month, 6-month tumor recurrence rate and recurrence rate of the same site 1 year after surgery. In addition, ERBT has fewer complications, and shorter catheter indentation time and hospital stay. The laser ERBT can also decrease tumor residual rate and re-TURBT.

## Introduction

Bladder cancer ranks tenth among all tumors in the world and is the sixth most common cancer and the ninth leading cause of cancer death among men^[1]^. Bladder cancer is classified as nonmuscle-invasive bladder cancer (NMIBC) or muscle-invasive bladder cancer (MIBC) depending on whether tumour cells have invaded the bladder wall muscle, and NMIBC accounts for about 75% of bladder cancer^[2]^.

Conventional transurethral resection of bladder tumor (cTURBT) using piecemeal resection strategy is the most important method for the diagnosis, treatment, and staging of NMIBC^[2,3]^. However, the piecemeal resection strategy may result in tumor implantation, tumor cell release into the bloodstream, low presence of detrusor muscle (DM) in the specimen and high residual tumor rate after initial TURBT^[4-6]^. It is believed that the absence of DM in specimens was associated with residual disease, early recurrence and tumor understaging^[2,7]^. Therefore, en bloc transurethral resection of bladder tumor (ERBT) has been widely used in clinic because ERBT can achieve good prognosis by providing a complete resection and preserving the integrity of specimens which contain detrusor muscle^[8-10]^.

In recent years, more and more studies that compared ERBT with cTURBT were published. However, the efficacy and safety of ERBT have not been conclusional. This article aimed to perform an updated systematic review and meta-analysis of randomized controlled trials (RCTs) on pathological outcomes, surgical safety and prognosis of ERBT versus cTURBT for NMIBC.

## Methods

## Search strategy

The meta-analysis was registered at PROSPERO. RCTs in which language was restricted to English were searched in the databases of Pubmed, Embase, Clinicaltrials.gov, and Cochrane. Search terms include “En bloc transurethral resection of bladder tumor,” “eTURB,” “ERBT,” “conventional transurethral resection of bladder tumor,” “cTURBT,” “nonmuscle-invasive bladder cancer,” “NMIBC” and related expressions. The references of the relevant articles were also reviewed. Only RCTs were included in the meta-analysis after all studies were browsed by two independent reviewers. The work has been reported in line with PRISMA (Preferred Reporting Items for Systematic Reviews and Meta-Analyses)^[11]^ and AMSTAR (Assessing the methodological quality of systematic reviews)^[12]^ Guidelines.

## Criteria for selection

Inclusion criteria: (1) RCTs that compared ERBT with cTURBT treatment for NMIBC; (2) RCTs should involve effective data; and (3) the full text of the RCT was available.

Excluded criteria: Reviews, non-RCTs, comments, case reports, recommendations, letters, ongoing trials, protocols, and studies lacking of applicable data.

## Data extraction

Two researchers extracted data independently such as first author, year, country, trial design, age, gender, surgical approach, sample size, tumor size, tumor number, tumor location, T stage, grading, and adjuvant therapy. The outcomes researched in this meta-analysis included: resected tissue contained detrusor muscle, tumor residual, bladder perforation, obturator nerve reflex, re-TURB, catheter indwelling time, hospital days, bleeding rate (bladder wash for more than 48 h, repeat cystoscopy, or blood transfusion), hemoglobin deficit (The difference between preoperative hemoglobin and postoperative hemoglobin), urethral stricture, resection time, operative time, the recurrence rate of the same site 1 year after surgery and 3-month, 6-month, 1-year, 2-year, 3-year tumor recurrence rate, and the risk of progression at 12 month. In instances where there were two distinct surgical approaches within the same RCTs, two sets of data were collected. In some RCTs included in this study, we estimated the sample mean and standard deviation from the sample size, median, and extremes according to Luo’s^[13]^ and Wan’s study^[14]^.

## Quality assessment

We used the Cochrane risk of bias tool to assess the risk of bias of the retrieved RCTs^[15]^. The quality items were random sequence generation, allocation concealment, blinding of participants and personnel, incomplete outcome data, and selective outcome reporting. The discussion among all reviewers was performed to resolve any uncertainties about the quality assessment.

## Data analysis

Review Manager version 5.4 was employed to analyze the data. We used mean difference (MD) with the corresponding 95% confidence intervals (CI) to explain continuous data and odds ratio (OR) for dichotomous results. If *P*-value < 0.05, the result of statistics was remarkable. The inconsistency was analyzed by using *I*^2^ statistic that mirrored the proportion of heterogeneity in data analysis. A random effect model would be used for results where the *I*^2^ value > 50% and has significant heterogeneity. Otherwise, fixed effect model was applicated. Publication bias was assessed with a funnel plot.

## Sensitivity analysis and Subgroup analysis

A sensitivity analysis was performed by excluding trials recruiting participants with particular conditions or trials with characteristics that were different from those in the other trials. Subgroup analyses was performed according to the prespecified factors: different techniques used in operation (laser and non-laser).

## Trial sequential analysis

Trial sequential analysis (TSA) was performed using Trial Sequential Analysis software version 0.9.5.10 beta. TSA was set to maintain the overall risk of type I error of 5% and 20% level of type II error (a power of 80%) and report the information size, an estimate of optimum sample size for statistical inference from a meta-analysis, after considering the heterogeneity of the included studies. When the cumulative Z-curve crossed the trial sequential monitoring boundary or exceeded the required information size line, it was considered to be an indicator of sufficient and firm evidence, with no further studies required. Otherwise, additional studies were needed.

## Rating certainty of evidence

The quality of the evidence was rated by the Grading of Recommendations, Assessment, Development and Evaluation (GRADE) approach^[16]^. We used the web version GRADEpro (https://www.gradepro.org/) to assess the certainty of evidence from the results of our meta-analysis.

## Results

### Characteristics of the individual studies

**Figure 1.**
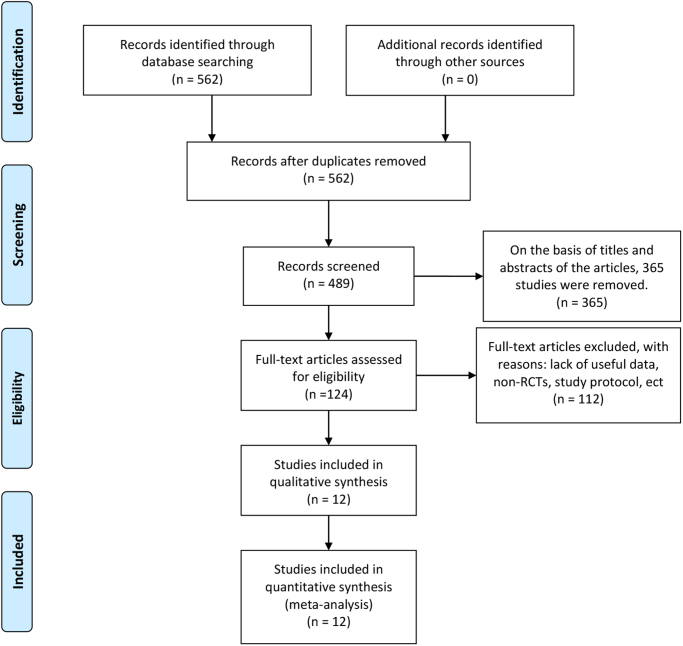
Flowchart of study inclusion.

A total of 686 articles were identified according to the searching terms. Finally, 12 RCTs^[17-28]^ that included 2432 participants (ERBT group: 1212 participants vs. cTURBT group: 1220 participants) were included in our meta-analysis (Fig. 1) according to inclusion and exclusion criteria. The characteristics of 12 selected RCTs are revealed in Table 1. The characteristics of bladder tumors are revealed in Table 2.

**Table 1:** Characteristics of studies and patients

Study, year, country	Trial design	Age (mean, SD)		Gender (m/f)		Surgical approach		Sample size		Inclusion criteria	Exclusion criteria	Number of re-TURBT	
		ERBT	cTURBT	ERBT	cTURBT	ERBT	cTURBT	ERBT	cTURBT			ERBT	cTURBT
**Andrea 2023 Austria**	RCT	65.89, 3.15	69.85, 2.97	133/45	140/39	Monopolar bipolar laser fiber	Monopolar bipolar laser fiber	178	179	Primary papillary NMIBC (stage cTa or cT1, 1–3 cm, ≤ 3 tumors)	Synchronous or a history of UTUC, life expectancy of <1 year, and pregnancy	26	34
**Badawy 2022 Egypt**	RCT	64.53, 8.48	61.83, 7.47	55/5	57/3	2-micron continue-wave laser	NA	60	60	Primary urinary bladder tumors (<4 cm)	> 4 cm, broad bases, or in the anterior wall or bladder dome. Recurrent cases and cases with advanced stage.		
**Balan 2018 Romania**	RCT	64.7, NA	66.1, NA	NA	NA	bipolar	Monopolar	45	45	Papillary bladder tumors (1–3 cm)	Solid sessile tumors, lesions located in the bladder neck area and tumors involving the ureteral orifice		
**Chen 2014 China**	RCT	62.23, 13.68	60.95, 14.53	54/17	51/20	2-μm continue-wave laser	NA	71	71	primary NMIBC	Patients with serious heart, lung or brain conditions. muscle-invasive tumor		
**Fan 2021 China**	RCT	59.95, 2.94	56.95, 2.94	96/20	87/30	Green-light laser	NA	116	117	Primary NMIBC (<3 cm)	Recurrent bladder cancer, ≥ 3-cm tumor		
**Gakis 2020 Germany**	RCT	66.8, 11.1	70.2, 12.4	45/11	47/12	HybridKnife	NA	56	59	A (newly diagnosed or recurrent) bladder tumour on cystoscopy	≤0.5 cm, muscle invasive tumour, > 5 tumour lesions, tumours too extensive to be resected and retrieved in one piece	19	23
**Gallioli 2022 Spain**	RCT	71.87, 3.63	72.89, 2.77	109/31	91/17	Monopolar Bipolar thulium laser	Monopolar bipolar	140	108	Primary or recurrent BC (≤ 3 cm, ≤ 3 tumors)	MIBC, ureteral involvement, and/or nodal/metastatic extension		
**Hashem 2021 Egypt**	RCT	60.4, 11.9	61.1, 11.3	37/13	39/11	Holmium laser	Monopolar	50	50	NMIBC	History of UTUC, anteriorly located bladder tumors, and/or tumors >5 lesions and/or >5 cm.	3	13
**Huang (1) 2015 China**	RCT	58.31, 6.13	57.87, 4.99	50/20	48/22	2-μm continue-wave laser	Monopolar	70	70	Primary NMIBC	Muscle invasive bladder tumors, recurrent tumors, distant metastases, or upper urinary tract tumors		
**Huang (2) 2015 China**	RCT	59.97, 5.75	57.87, 4.99	45/25	48/22	Holmium laser	Monopolar	70	70				
**Liu 2013 China**	RCT	67.1, 8.3	66.3, 9.8	46/18	40/16	2-μm continue-wave laser	Monopolar	64	56	Newly diagnosed NMIBC	Urothelial papillomas, muscle-invasive bladder tumors, *in situ* carcinomas or upper urinary tract tumours		
**Teoh 2024 China**	RCT	70.04, 2.86	69.14, 3.27	108/35	110/23	Bipolar	Bipolar	143	133	NMIBC (≤ 3 cm)	> 3 cm, MIBC, presence or prior history of UTUC	27	18
**Zhang 2015 China**	RCT	NA	NA	70/79	79/64	2-μm continue-wave laser	Bipolar	149	143	Newly diagnosed Ta or T1 bladder cancer	Extravesical tumor extension, lymph node metastasis, or adjacent organ invasion	17	27

RCT, randomized controlled trial; ERBT, en-bloc resection of bladder tumor; cTURBT, conventional transurethral resection of bladder tumor; UTUC, upper urinary tract urothelial cancer.

**Table 2:** Characteristics of tumors

Study, year, country	Tumor size (mean, SD) (cm)		Tumor number (single/multiple)		Tumor location (Lateral/other)		T stage (Tis/ Ta/T1)		Grade (PUNLMP/ low/high)		Adjuvant therapy
	ERBT	cTURBT	ERBT	cTURBT	ERBT	cTURBT	ERBT	cTURBT	ERBT	cTURBT	
**Andrea 2023 Austria**	1.87, 0.19	1.7, 0.28	NA	NA	134/85	133/100	2/142/56	4/146/57	8/129/68	5/117/93	NA
**Badawy 2022 Egypt**	1.25, 1.06	1.40, 0.65	58/2	55/5	40/20	31/29	0/18/34	0/24/30	15/37/8^a^	24/30/6^a^	D
**Balan 2018 Romania**	1.82, NA	1.69, NA	17/28	19/26	NA	NA	0/24/21	0/23/22	NA	NA	E and BCG
**Chen 2014 China**	2.6, 1.4	2.3, 1.2	NA	NA	73/55	63/58	3/43/25	1/55/15	5/43/23	9/45/17	E
**Fan 2021 China**	1.48, 0.06	1.5, 0.2	87/29	92/25	61/55	7/70	0/91/25	0/104/13	5/74/37	10/77/30	P
**Gakis 2020 Germany**	NA	NA	NA	NA	NA	NA	0/50/6	0/42/17	20/25/9/2^b^	15/23/17/4^b^	M and/or BCG
**Gallioli 2022 Spain**	NA	NA	NA	NA	NA	NA	2/76/15	2/39/9	70/55/4^c^	56/41/4^c^	M or E
**Hashem 2021 Egypt**	3.2, 1.1	2.9, 1.4	33/17	28/22	18/11	13/13	0/2/42	0/3/36	0/28/16	1/26/22	E
**Huang (1) 2015 China**	1.63, 0.32	1.53, 0.20	NA	NA	23/47	25/45	7/40/23	8/35/27	20/40/10	18/46/6	E
**Huang (2) 2015 China**	1.58, 0.51	1.53, 0.20	NA	NA	28/42	25/45	5/37/28	8/35/27	15/48/7	18/46/6	E
**Liu 2013 China**	1.31, 0.23	1.28, 0.31	NA	NA	24/40	21/35	0/37/27	0/34/22	11/46/7	10/41/5	E
**Teoh 2024 China**	1.5, 0.19	1.95, 0.19	97/46	86/47	86/57	69/64	2/110/31	3/112/18	6/92/45	3/93/37	M
**Zhang 2015 China**	NA	NA	77/72	78/65	NA	NA	0/106/43	0/107/36	87/54/8^d^	75/60/8^d^	E

ERBT, en-bloc resection of bladder tumor; cTURBT, conventional transurethral resection of bladder tumor; NA, not available; PUNLMP, papillary urothelial; neoplasms of low malignant potential; D, doxorubicin; E, epirubicin; BCG, Bacille–Calmette Guerin; P, pirarubicn; M: mitomycin.

^a^ Low/high/invasive TCC according to EAU 2022.

^b^ G1/G2/G3/GX according to WHO 1973.

^c^ Low/high/Tis according to EAU 2018.

^d^ G0/G1/G2 according to WHO 1973.

## Quality of the RCTs

**Figure 2.**
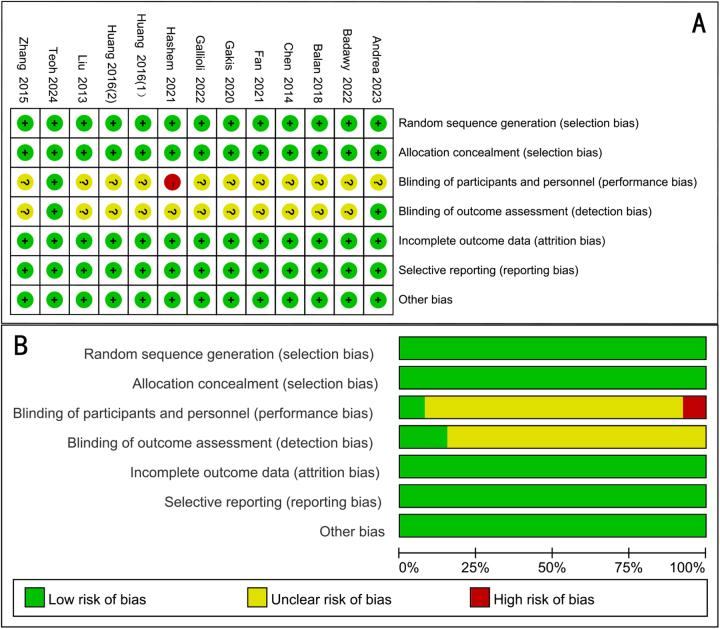
Assessment of randomized study quality. (A) Risk of bias summary, (B) risk of bias graph.

All of the 12 articles included in the meta-analysis were RCTs. The assessment of risk of bias of studies was showed in Fig. 2. The funnel plot showed that publication bias were found in obturator nerve reflex, bladder perforation, and operative time (Supplement Figure 1 http://links.lww.com/JS9/D913). And in subgroup analysis, there was an asymmetry for nonlaser ERBT group (Supplement Figure 1 http://links.lww.com/JS9/D913).

## Result of meta-analysis

### Detrusor muscle

**Figure 3.**
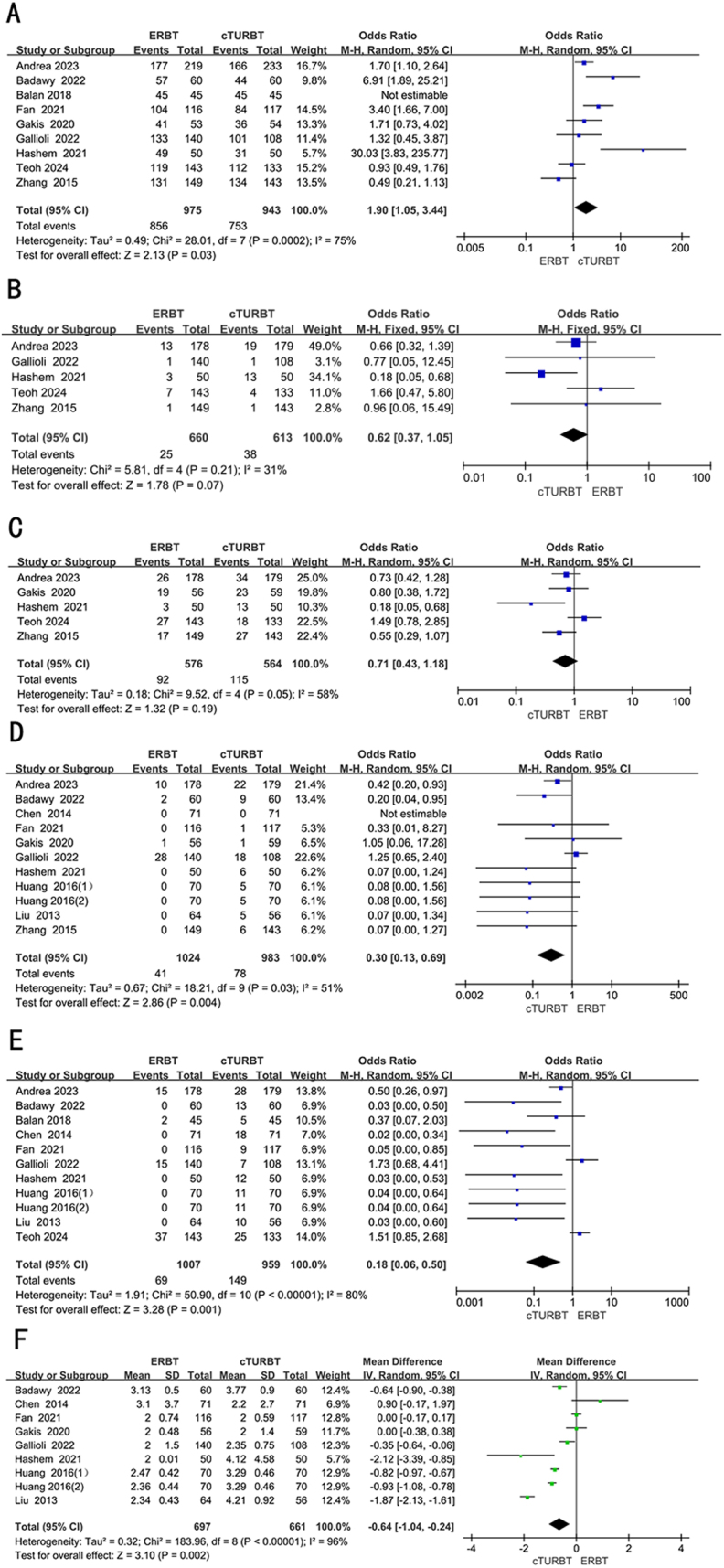
Forest plots of (A) detrusor muscle presence, (B) residual tumor, (C) re-TURBT, (D) bladder perforation, (E) obturator nerve reflex, (F) catheter indwelling time.

Nine RCTs, involving 1918 patients, were adopted to analyze the resected tissue contained detrusor muscle. Employing a random-effects model, we assessed the difference in resected tissue contained detrusor muscle between two groups. The result revealed that the resected tissue contained detrusor muscle was significantly more in the ERBT group (OR 1.90; 95% CI 1.05 to 3.44; *P* = 0.03; *I*^2^ = 75%) (Fig. 3A).

According to the techniques used in operation and the inclusion criteria for our studies, we divided the included studies into two groups for subgroup analyses: five RCTs were laser ERBT group and four RCTs were nonlaser ERBT group. The result revealed that the resected tissue contained detrusor muscle was significantly more in the laser ERBT groups (OR 2.74; 95% CI 1.05 to 7.10; *P* = 0.04; *I*^2^ = 83%) compared with cTURBT group (OR 1.18; 95% CI 0.75 to 1.88; *P* = 0.47; *I*^2^ = 0%) (Supplement Figure 2A http://links.lww.com/JS9/D914).

## Residual tumor

Five RCTs full of 1273 patients were applied to assess the residual tumor. A fixed-effects model was utilized to evaluate the difference in residual tumor between two groups. The result revealed that the residual tumor was similar between two groups (OR 0.62; 95% CI 0.37 to 1.05; *P* = 0.07; *I*^2^ = 31%) (Fig. 3B).

According to the techniques used in operation and the inclusion criteria for our studies, we divided the included studies into two groups for subgroup analyses: three RCTs were laser ERBT group and three RCTs were nonlaser ERBT group. The result revealed that the rate of re-TURB was significantly less in the laser ERBT groups (OR 0.48; 95% CI 0.26 to 0.89; *P* = 0.02; *I*^2^ = 34%) compared with cTURBT group (OR 1.46; 95% CI 0.47 to 4.55; *P* = 0.51; *I*^2^ = 0%) (Supplement Figure 2B http://links.lww.com/JS9/D914).

## Re-TURBT

Five RCTs, involving 1140 patients, were adopted to analyze the re-TURB. Employing a random-effects model, we assessed the difference in time to re-TURB between two groups. The result revealed that the rate of re-TURB was similar between two groups (OR 0.71; 95% CI 0.43 to 1.18; *P* = 0.19; *I*^2^ = 58%) (Fig. 3C).

According to the techniques used in operation and the inclusion criteria for our studies, we divided the included studies into two groups for subgroup analyses: three RCTs were laser ERBT group and two RCTs were nonlaser ERBT group. The result revealed that the rate of re-TURB was significantly less in the laser ERBT groups (OR 0.53; 95% CI 0.29 to 0.95; *P* = 0.03; *I*^2^ = 45%) compared with cTURBT group (OR 1.13; 95% CI 0.62 to 2.06; *P* = 0.69; *I*^2^ = 31%) (Supplement Figure 2C http://links.lww.com/JS9/D914).

## Bladder perforation

Ten RCTs, involving 1937 patients, were adopted to analyze the bladder perforation. Employing a random-effects model, we assessed the difference in bladder perforation between two groups. The result revealed that the bladder perforation was significantly less in the ERBT groups (OR 0.30; 95% CI 0.13 to 0.69; *P* = 0.004; *I*^2^ = 51%) (Fig. 3D).

According to the techniques used in operation and the inclusion criteria for our studies, we divided the included studies into two groups for subgroup analyses: eight RCTs were laser ERBT group and two RCTs were nonlaser ERBT group. The result revealed that the bladder perforation was significantly less in the laser ERBT groups (OR 0.26; 95% CI 0.14 to 0.47; *P*<0.0001; *I*^2^ = 0%) compared with cTURBT group (OR 1.24; 95% CI 0.66 to 2.34; *P* = 0.66; *I*^2^ = 0%) (Supplement Figure 2D http://links.lww.com/JS9/D914).

## Obturator nerve reflex

Ten RCTs full of 1896 patients were applied to assess the obturator nerve reflex. A random-effects model was utilized to evaluate the difference in obturator nerve reflex between two groups. The forest plot illustrated a significant decrease in the obturator nerve reflex within the ERBT group compared to the cTURBT group (OR 0.18; 95% CI 0.06 to 0.50; *P* = 0.001; *I*^2^ = 80%) (Fig. 3E).

According to the techniques used in operation and the inclusion criteria for our studies, we divided the included studies into two groups for subgroup analyses: seven RCTs were laser ERBT group and three RCTs were nonlaser ERBT group. The result revealed that the obturator nerve reflex was significantly less in the laser ERBT groups (OR 0.06; 95% CI 0.01 to 0.24; *P* < 0.0001; *I*^2^ = 67%) compared with cTURBT group (OR 1.34; 95% CI 0.74 to 2.44; *P* = 0.33; *I*^2^ = 23%) (Supplement Figure 2E http://links.lww.com/JS9/D914).

## Catheter indwelling time

Eight RCTs full of 1288 patients were applied to assess the catheter indwelling time. A random-effects model was utilized to evaluate the difference in catheter indwelling time between two groups. The forest plot illustrated a significant decrease in the catheter indwelling time within the ERBT group compared to the cTURBT group [weighted mean difference (WMD) − 0.64; 95% CI −1.04 to **−0**.24; *P* = 0.002; *I*^2^ = 96%] (Fig. 3F).

According to the techniques used in operation and the inclusion criteria for our studies, we divided the included studies into two groups for subgroup analyses: six RCTs were laser ERBT group and two RCTs were nonlaser ERBT group. The result revealed that the catheter indwelling time was significantly less in the laser ERBT groups (WMD −0.78; 95% CI −1.26 to **−0**.31; *P* = 0.001; *I*^2^ = 96%) compared with cTURBT group (WMD −0.20; 95% CI −0.54 to 0.14; *P* = 0.25; *I*^2^ = 52%) (Supplement Figure 3A http://links.lww.com/JS9/D915).

## Bleeding rate

**Figure 4.**
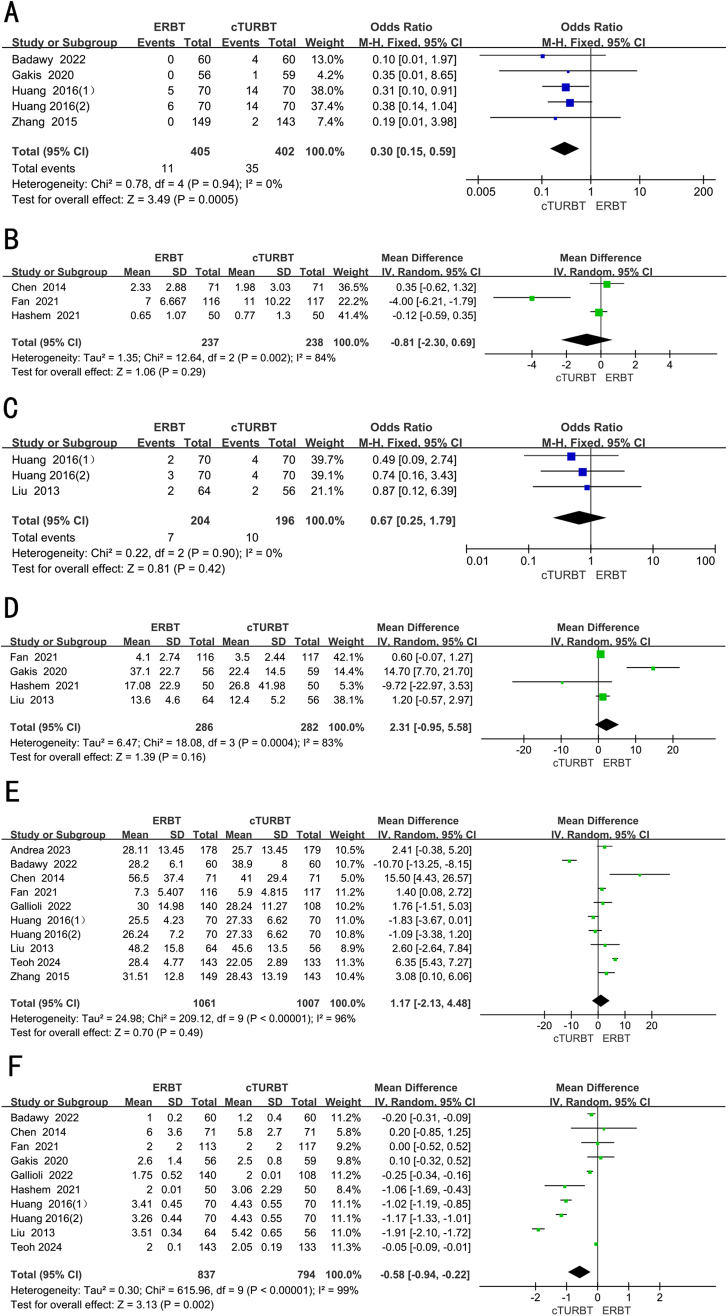
Forest plots of (A) bleeding rate, (B) hemoglobin deficit, (C) urethral stricture, (D) resection time, (E) operative time, (F) hospital days.

Four RCTs full of 737 patients were applied to assess the bleeding rate. A fixed-effects model was utilized to evaluate the difference in bleeding rate between two groups. The forest plot illustrated a significant decrease in the bleeding rate within the ERBT group compared to the cTURBT group (OR 0.30; 95% CI 0.15 to 0.59; *P* = 0.0005; *I*^2^ = 0%) (Fig. 4A).

## Hemoglobin deficit

Three RCTs, involving 475 patients, were adopted to analyze the hemoglobin deficit. Employing a random-effects model, we assessed the difference in decreased hemoglobin between two groups. The result revealed that the hemoglobin deficit was similar between two groups (WMD −0.81; 95% CI −2.30 to 0.69; *P* = 0.29; *I*^2^ = 84%) (Fig. 4B).

## Urethral stricture

Two RCTs full of 330 patients were applied to assess the urethral stricture. A fixed-effects model was utilized to evaluate the difference in urethral stenosis between two groups. The forest plot illustrated that the evidence of urethral stricture was comparable between two groups (OR 0.67; 95% CI 0.25 to 1.79; *P* = 0.42; *I*^2^ = 0%) (Fig. 4C).

## Resection time

Four RCTs, involving 568 patients, were adopted to analyze the resection time. Employing a random-effects model, we assessed the difference in resection time between two groups. The result revealed that there was no difference in resection time between two groups (WMD 2.31; 95% CI −0.95 to 5.58; *P* = 0.16; *I*^2^ = 83%) (Fig. 4D).

## Operative time

Nine RCTs full of 1998 patients were applied to assess the operative time. A random-effects model was utilized to evaluate the difference in operative time between two groups. The forest plot illustrated that there was no difference in operative time between two groups (WMD 1.17; 95% CI −2.13 to 4.48; *P* = 0.49; *I*^2^ = 96%) (Fig. 4E).

According to the techniques used in operation and the inclusion criteria for our studies, we divided the included studies into two groups for subgroup analyses: seven RCTs were laser ERBT group and two RCTs were nonlaser ERBT group. Whether in the laser (WMD 0.20; 95% CI −3.09 to 3.49; *P* = 0.90; *I*^2^ = 92%) or non-laser ERBT group (WMD 1.76; 95% CI −0.55 to 4.07; *P* = 0.14; *I*^2^ = 0%), there was no differences in operative time between two groups (Supplement Figure 3B http://links.lww.com/JS9/D915).

## Hospital days

Nine RCTs, involving 1561 patients, were adopted to analyze the length of hospital days. Employing a random-effects model, we assessed the difference in the length of hospital days between two groups. The result revealed that the length of hospital days was significantly short in the ERBT groups (WMD −0.58; 95% CI −0.94 to −0.22; *P* = 0.002; *I*^2^ = 99%) (Fig. 4F).

According to the techniques used in operation and the inclusion criteria for our studies, we divided the included studies into two groups for subgroup analyses: six RCTs were laser ERBT group and two RCTs were nonlaser ERBT group. The result revealed that the length of hospital days was significantly less in the laser ERBT groups (WMD −0.79.53; 95% CI −1.36 to **−0**.22; *P* = 0.006; *I*^2^ = 98%) compared with cTURBT group (WMD −0.11; 95% CI −0.29 to 0.06; *P* = 0.21; *I*^2^ = 89%) (Supplement Figure 3C http://links.lww.com/JS9/D915).

## The recurrence rate of the same site 1 year after surgery

**Figure 5.**
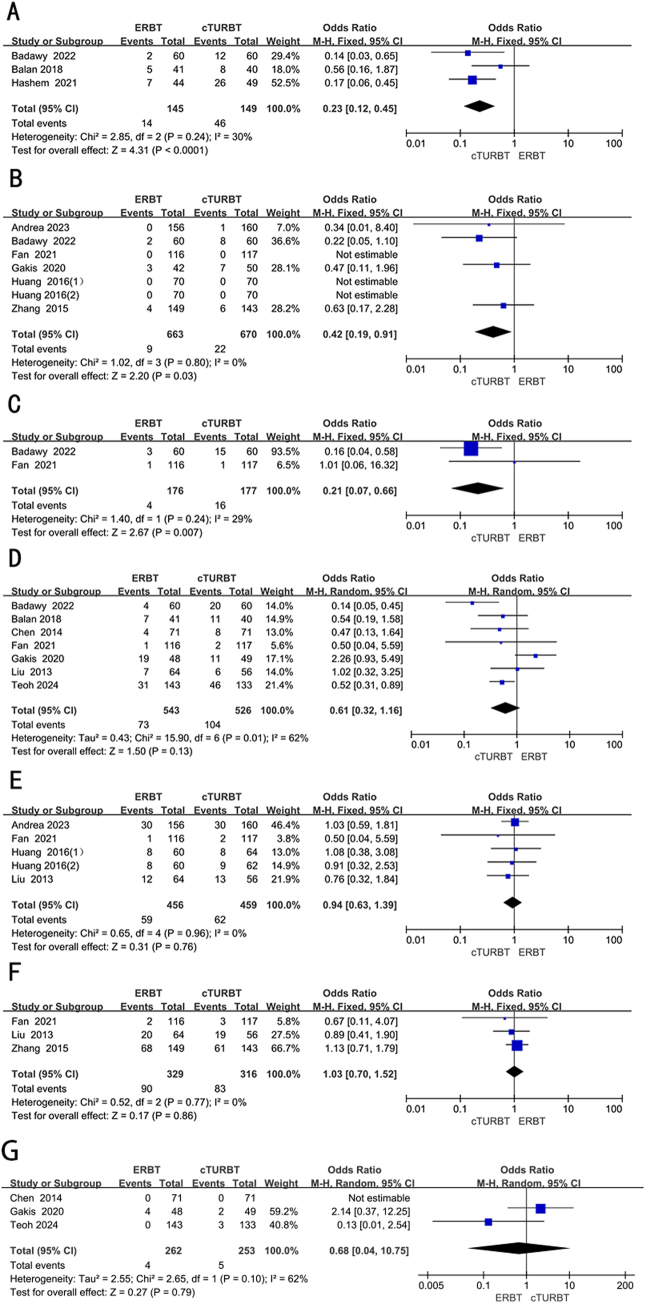
Forest plots of (A) the recurrence rate of the same site 1 year after surgery, (B) 3-month tumor recurrence rate, (C) 6-month tumor recurrence rate, (D) 1-year tumor recurrence rate, (E) 2-year tumor recurrence rate, (F) 3-year tumor recurrence rate, (G) the risk of progression at 12 months.

Three RCTs, involving 294 patients, were adopted to analyze the recurrence rate of the same site 1 year after surgery. Employing a fixed-effects model, we assessed the difference in the recurrence rate of the same site 1 year after surgery between two groups. The result revealed that the recurrence rate of the same site 1 year after surgery was significantly lower in the ERBT group (OR 0.23; 95% CI 0.12 to 0.45; *P* < 0.0001; *I*^2^ = 30%) (Fig. 5A).

## 3-Month tumor recurrence rate

Six RCTs full of 1263 patients were applied to assess the 3-month tumor recurrence rate. A random-effects model was utilized to evaluate the difference in 3 months tumor recurrence rate between two groups. The forest plot illustrated a significant decrease in the 3 months tumor recurrence rate within the ERBT group compared to the cTURBT group (OR 0.42; 95% CI 0.19 to 0.91; *P* = 0.03; *I*^2^ = 0%) (Fig. 5B).

## 6-Month tumor recurrence rate

Two RCTs, involving 343 patients, were adopted to analyze the 6-month tumor recurrence rate. Employing a fixed-effects model, we assessed the difference in the 6-month tumor recurrence rate between two groups. The result revealed that the 6-month tumor recurrence rate was significantly lower in the ERBT group (OR 0.21; 95% CI 0.07 to 0.66; *P* = 0.007; *I*^2^ = 29%) (Fig. 5C).

## 1-Year tumor recurrence rate

Seven RCTs full of 1069 patients were applied to assess the 1-year tumor recurrence rate. A random-effects model was utilized to evaluate the difference in 1 year tumor recurrence rate between two groups. The forest plot illustrated the 1 year tumor recurrence rate was comparable between two groups (OR 0.61; 95% CI 0.32 to 1.16; *P* = 0.13; *I*^2^ = 62%) (Fig. 5D).

According to the techniques used in operation and the inclusion criteria for our studies, we divided the included studies into two groups for subgroup analyses: four RCTs were laser ERBT group and three RCTs were nonlaser ERBT group. Whether in the laser (OR 0.42; 95% CI 0.16 to 1.08; *P* = 0.07; *I*^2^ = 48%) or nonlaser ERBT group (OR 0.85; 95% CI 0.33 to 2.18; *P* = 0.73; *I*^2^ = 75%), there was no differences in 1-year tumor recurrence rate between two groups (Supplement Figure 3D http://links.lww.com/JS9/D915).

## 2-Year tumor recurrence rate

Four RCTs, involving 655 patients, were adopted to analyze the tumor recurrence rate of the 2-year after surgery. Employing a fixed-effects model, we assessed the difference in the 2 years tumor recurrence rate between two groups. The result revealed that the 2-year tumor recurrence rate was comparable between two groups (OR 0.94; 95% CI 0.63 to 1.39; *P* = 0.76; *I*^2^ = 0%) (Fig. 5E).

## 3-Year tumor recurrence rate

Three RCTs full of 645 patients were applied to assess the 3-year tumor recurrence rate. A random-effects model was utilized to evaluate the difference in 3-year tumor recurrence rate between two groups. The forest plot illustrated a significant decrease in the 3-year tumor recurrence rate within the ERBT group compared to the cTURBT group (OR 1.03; 95% CI 0.70 to 1.52; *P* = 0.86; *I*^2^ = 0%) (Fig. 5F).

## The risk of progression at 12 months

Three RCTs full of 515 patients were applied to assess the risk of progression at 12 month. A random-effects model was utilized to evaluate the difference in the risk of progression at 12 months between two groups. The result revealed that the risk of progression at 12 months was comparable between two groups (OR 0.68; 95% CI 0.04 to 10.75; *P* = 0.79; *I*^2^ = 62%) (Fig. 5G).

## Results of sensitivity analysis

We performed a sensitivity analysis to examine the stability of the outcomes. For hemoglobin deficit, we exclude a relative outlier, the results showed that *I*^2^ changed from 84 to 0 (Supplement Figure 4A http://links.lww.com/JS9/D916). It indicated that the heterogeneity was mainly due to the study by Fan in 2021. For resection time, we exclude a relative outlier, the result showed that *I*^2^ changed from 83 to 27, indicating that the heterogeneity was mainly due to the study by Gakis in 2020 (Supplement Figure 4B http://links.lww.com/JS9/D916).

## TSA

**Figure 6.**
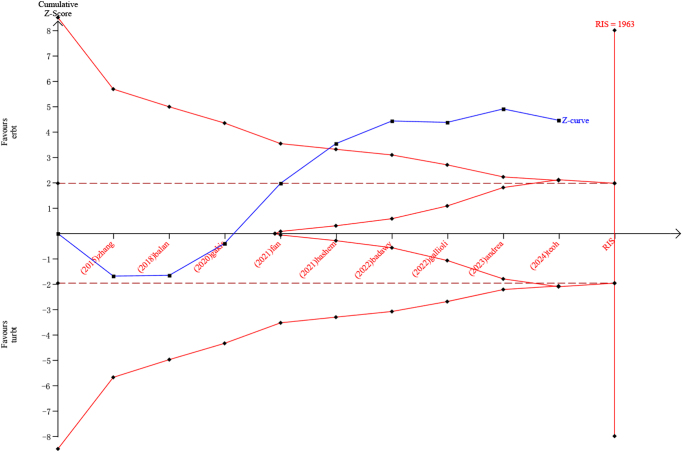
Trial sequential analysis for detrusor muscle presence.

Figure 6 shows the results of the TSA using a 20% RRR threshold. The Z-curve crosses the superiority boundary after five RCTs, which indicates that the available evidence is sufficient to suggest that ERBT compared with cTURBT would increase the OR of detrusor muscle presence by 20% in specimens. The required sample size was determined (Fig. 6).

## Grading of evidence

According to the GRADE approach, we collected evidence from our systematic review to summarize the findings (Table 3).

**Table 3:** Assessment sheet of overall quality of evidence by GRADE guidelines

**Certainty assessment**							**№ of patients**		**Effect**		**Certainty**	**Importance**
**№ of studies**	**Study design**	**Risk of bias**	**Inconsistency**	**Indirectness**	**Imprecision**	**Other considerations**	**ERBT**	**cTURBT**	**Relative(95% CI)**	**Absolute(95% CI)**		
**Detrusor muscle**												
9	randomised trials	not serious	very serious^a^	not serious	not serious	none	856/975 (87.8%)	753/943 (79.9%)	**OR 1.90**(1.05 to 3.44)	**84 more per 1,000**(from 8 more to 133 more)	⊕⊕◯◯Low^a^	CRITICAL
**residual tumor**												
5	randomised trials	not serious	not serious	not serious	not serious	none	49/660 (7.4%)	73/613 (11.9%)	**OR 0.53**(0.35 to 0.78)	**52 fewer per 1,000**(from 74 fewer to 24 fewer)	⊕⊕⊕⊕High	CRITICAL
**bladder perforation**												
10	randomised trials	not serious	serious^b^	not serious	not serious	strong association	41/1024 (4.0%)	78/983 (7.9%)	**OR 0.30**(0.13 to 0.69)	**54 fewer per 1,000**(from 68 fewer to 23 fewer)	⊕⊕⊕⊕High^b^	IMPORTANT
**Obturator nerve reflex**												
10	randomised trials	not serious	very serious^a^	not serious	not serious	strong association	69/1007 (6.9%)	149/959 (15.5%)	**OR 0.18**(0.06 to 0.50)	**123 fewer per 1,000**(from 144 fewer to 71 fewer)	⊕⊕⊕◯Moderate^a^	IMPORTANT
**re-TURB**												
5	randomised trials	not serious	serious^b^	not serious	serious^c^	none	92/576 (16.0%)	115/564 (20.4%)	**OR 0.71**(0.43 to 1.18)	**50 fewer per 1,000**(from 105 fewer to 28 more)	⊕⊕◯◯Low^b,c^	IMPORTANT
**catheter dwell time**												
8	randomised trials	not serious	very serious^a^	not serious	not serious	none	697	661	-	MD **0.64 days lower**(1.04 lower to 0.24 lower)	⊕⊕◯◯Low^a^	IMPORTANT
**bleeding rate**												
4	randomised trials	not serious	not serious	not serious	not serious	strong association	11/405 (2.7%)	35/402 (8.7%)	**OR 0.30**(0.15 to 0.59)	**59 fewer per 1,000**(from 73 fewer to 34 fewer)	⊕⊕⊕⊕High	IMPORTANT
**hemoglobin deficit**												
3	randomised trials	not serious	very serious^a^	not serious	serious^c^	none	237	238	-	MD **0.17 g/mL lower**(0.58 lower to 0.24 higher)	⊕◯◯◯Very low^a,c^	IMPORTANT
**urethral stricture**												
2	randomised trials	not serious	not serious	not serious	serious^c^	none	7/204 (3.4%)	10/196 (5.1%)	**OR 0.67**(0.25 to 1.79)	**16 fewer per 1,000**(from 38 fewer to 37 more)	⊕⊕⊕◯Moderate^c^	IMPORTANT
**resection time**												
4	randomised trials	not serious	very serious^a^	not serious	serious^c^	none	230	223	-	MD **0.7 min higher**(0.42 lower to 1.81 higher)	⊕◯◯◯Very low^a,c^	IMPORTANT
**operative time**												
9	randomised trials	not serious	very serious^a^	not serious	serious^c^	none	1061	1007	-	MD **1.17 min higher**(2.13 lower to 4.48 higher)	⊕◯◯◯Very low^a,c^	IMPORTANT
**length of hospital stay**												
9	randomised trials	not serious	very serious^a^	not serious	not serious	none	837	794	-	MD **0.58 days lower**(0.94 lower to 0.22 lower)	⊕⊕◯◯Low^a^	IMPORTANT
**Recurrence rate of the same site 1 year after surgery**												
3	randomised trials	not serious	not serious	not serious	serious^d^	strong association	14/145 (9.7%)	46/149 (30.9%)	**OR 0.23**(0.12 to 0.45)	**216 fewer per 1,000**(from 258 fewer to 141 fewer)	⊕⊕⊕⊕High^d^	CRITICAL
**3-M tumor recurrence raye**												
6	randomised trials	not serious	not serious	not serious	not serious	strong association	9/663 (1.4%)	22/670 (3.3%)	**OR 0.42**(0.19 to 0.91)	**19 fewer per 1,000**(from 26 fewer to 3 fewer)	⊕⊕⊕⊕High	CRITICAL
**6-M tumor recurrence raye**												
2	randomised trials	not serious	not serious	not serious	not serious	strong association	4/176 (2.3%)	16/177 (9.0%)	**OR 0.21**(0.07 to 0.66)	**70 fewer per 1,000**(from 83 fewer to 29 fewer)	⊕⊕⊕⊕High	CRITICAL
**1-Y tumor recurrence rate**												
7	randomised trials	not serious	serious^b^	not serious	serious^c^	none	73/543 (13.4%)	104/526 (19.8%)	**OR 0.61**(0.32 to 1.16)	**67 fewer per 1,000**(from 125 fewer to 25 more)	⊕⊕◯◯Low^b,c^	CRITICAL
**2-Y tumor recurrence rate**												
4	randomised trials	not serious	not serious	not serious	serious^c^	none	59/456 (12.9%)	62/459 (13.5%)	**OR 0.94**(0.63 to 1.39)	**7 fewer per 1,000**(from 46 fewer to 43 more)	⊕⊕⊕◯Moderate^c^	CRITICAL
**3-Y tumor recurrence rate**												
3	randomised trials	not serious	not serious	not serious	serious^c^	none	90/329 (27.4%)	83/316 (26.3%)	**OR 1.03**(0.70 to 1.52)	**6 more per 1,000**(from 63 fewer to 89 more)	⊕⊕⊕◯Moderate^c^	CRITICAL		
**The risk of progression at 12 month**												
3	randomised trials	not serious	not serious	not serious	serious^c^	none	4/262 (1.5%)	5/253 (2.0%)	**OR 0.68**(0.04 to 10.75)	**6 fewer per 1,000**(from 19 fewer to 158 more)	⊕⊕⊕◯Moderate^a^	CRITICAL

CI, confidence interval; MD, mean difference; OR, odds ratio.

^a^ There is significant heterogeneity. *I*^2^ ≥ 75%.

^b^ There is significant heterogeneity. 50% < *I*^2^ < 75%.

^c^ 95% CI across the equivalence line.

^d^ Sample size <300.

## Discussion

cTURBT is the gold standard for treating NMIBC^[29]^, the presence of detrusor muscle in the pathological specimens is the most reliable qualified indicator of adequate resection^[30]^. Herr *et al* also reported that complete resection, detrusor muscle presence in the specimen and same-site recurrence rate after previous TURBT were the ways to assess the quality of TURBT^[31]^. However, some studies showed that detrusor muscle was present in 80% of the patients who underwent cTURBT^[6,32]^. cTURBT was also associated with obturator nerve reflex and bladder perforation^[33,34]^. More and more researches demonstrated that fewer complications and more high-quality specimens were present in ERBT^[17-21]^. In the present meta-analysis, we comprehensively reviewed the all past RCTs to evaluate the efficacy and safety of ERBT and cTURBT for the patients with NMIBC.

This meta-analysis found that the rate of identification of detrusor muscle in specimens was higher in ERBT group, the rate of re-TURBT and residual tumor rate was comparable between two groups. DM presence in the specimen is a well-recognized indicator of high-quality resection, and is associated with better staging, risk stratification, and outcomes^[32,35]^. The ERBT preserves the structural integrity of the tumor tissue, including the lamina propria and detrusor muscle, thereby significantly improving the accuracy of pathological staging. A retrospective single-center study demonstrated that ERBT enhanced the quality of resection, achieving detrusor muscle retrieval in over 90% of resected tumors^[36]^. The cTURBT involves piecemeal resection of the tumor, which may lead to incomplete resection, fragmentation of the tumor tissue, and dissemination of floating cancer cells. These issues can result in the absence of detrusor muscle in the specimen and are associated with a higher rate of tumor recurrence. Moreover, electrocautery is routinely employed in cTURBT, which often results in the formation of eschar on tumor fragments, complicating the histopathological evaluation of the specimen. So subgroup analysis showed that non-laser ERBT did not increase the rate of of detrusor muscle in specimens. As a consequence, distinguishing the detrusor muscle becomes challenging for cTURBT, potentially leading to an underestimation of the depth of tumor invasion^[37]^. Li’s meta-analysis of RCTs reported that there was no significant difference in detrusor muscle presence^[38]^. However, the rate of re-TURBT was comparable between two groups. This result is similar to Zhang’s meta-analysis but different from Li’s meta-analysis^[38,39]^. Re-TURBT is a second resection procedure performed following the initial TURBT, as recommended by the European Association of Urology (EAU) guidelines for patients diagnosed with high-risk NMIBC^[40]^. Almost all guidelines recommend re-TURBT if the initial resection is incomplete^[6]^. However, the incomplete resection in initial TURBT is not the only indication of re-TURBT. Therefore, the rate of re-TURBT is not necessarily correlated with the different TUR procedure. Therefore, although subgroup analysis showed a lower incidence of re-TURBT in the laser ERBT group, this result needs to be interpreted with more caution. There was no significant difference on residual tumor rate. However, the result of subgroup analysis showed that residual tumor rate was lower in laser ERBT group. The ERBT technique is executed through a precise incision adjacent to the tumor, extending down to the muscular layer, allowing for the complete resection of the lesion in a single piece. However, non-laser ERBT may affect the diagnosis of tissue incisal margin due to electrocautery, thus affecting the tumor residual rate.

For the perioperative outcomes, our study revealed that bladder perforation, obturator nerve reflex, catheter indwelling time, length of hospital stay, and bleeding rate were significantly lower in ERBT group than that in cTURBT group, especially in laser ERBT group, hemoglobin deficit, urethral stricture, resection time, and operative time were comparable between two groups. Bladder perforation can occur as a consequence of the obturator nerve reflex or thermal injury to the bladder wall^[41]^. TURBT may lead to electrical stimulation of the obturator nerve, which causes leg adductor muscle contraction and then bladder perforation. However, nonlaser ERBT could not decrease the rate of obturator nerve reflex and bladder perforation due to heat damage and current stimulation. Yang’s study also demonstrated that monopolar ERBT would not obviate obturator nerve reflex^[42]^. However, laser ERBT is characterized by shallow heat damage, the shorter contact time between the laser fiber and tumor tissue, the absence of current stimulation and better hemostatic effect^[18]^. Liu’s study also reported the same advantages in laser ERBT^[43]^. These advantages also lead to shorter catheter indwelling time and length of hospital stay. These are consistent with the results of many studies^[19,22,24,44]^. However, Li’s meta-analysis revealed that there were no significant differences in catheter indwelling time and length of hospital stay^[39]^. For hemoglobin deficit, urethral stricture, resection time, and operative time, the results were similar to the previous study^[39,45]^. Xu’s retrospective study also found nonsignificant difference between laser ERBT and cTURBT in operative time^[46]^. Gakis reported that certain locations, such as the dome, anterior bladder wall, or areas in close proximity to the urethral orifice, may pose challenges when addressed with this technique. Also a very high number of lesions to be resected may make a ERBT excessively time-consuming^[22]^. From a learning curve standpoint, early-career practitioners are expected to gain the most from ERBT in cases where the risk of obturator nerve reflex is particularly high, such as in the management of lateral wall tumors. However, given the limited number of studies addressing surgeon experience, the learning curve associated with en bloc resection remains poorly defined. In addition, the cost of the ERBT group is higher than that of the TURBT group, the cost-effectiveness is also one of the limitations of ERBT applications, but there is also little research on this and the patient perspective, which needs to be further investigated in prospective trials.

Our meta-analysis found that tumor recurrence rate 3 months after surgery, tumor recurrence rate 6 months after surgery, the recurrence rate of the same site 1 year after surgery were significantly lower in ERBT group, 1-year, 2-year, and 3-year tumor recurrence rate were comparable between the two groups. The implementation of ERBT effectively prevents the dissemination of cancerous debris and residual tumor at the tumor base, thereby reducing the recurrence rate and contributing to an improved short-term prognosis. Residual tumor tissue and the release and seeding of the tumor cells during piecemeal resection in cTURBT may lead to higher tumor recurrence rate^[39,45]^. Many previous studies reported that ERBT did not show a relative advantage over cTURBT in reoccurrence rate at 3 months, 6 months, 1 year, 2 years, and 3 years^[38,39,47-49]^. Wang’s meta-analysis demonstrated that lower rates of 3-month, 24-month recurrence rate and same-site recurrence rate were also observed in ERBT group^[45]^. At present, few studies have given data on tumor recurrence. Especially for 6-month tumor recurrence rate, only two studies reported the result, this result needs to be interpreted with more caution. Bladder cancer is a chronic disease and needs to be detected for a long time. Lack of long-term follow-up data limits the ability to fully assess the durability of the observed benefits and potential late complications associated with ERBT. And the resection technique alone is not the sole determinant of subsequent oncological outcomes. Other factors, such as tumor biology and postoperative intravesical therapies, must also be considered in the long-term management of disease recurrence and progression^[50]^. Therefore, more researches are needed to further reveal these results.

In the meta-analysis, we only included RCTs that compared ERBT with cTURBT treatment for patients with NMIBC. And compared to previous meta-analysis, our study encompassed a substantially larger cohort of patients and RCTs. However, it is essential to acknowledge certain limitations in our study. First, we included different techniques used in operation such as different types of lasers, electrocauterization, and hybrid knife. Second, the presence of heterogeneity and publication bias across multiple outcomes significantly diminished the overall quality of the evidence, these results need to be interpreted with more caution. Third, for some results, only a few studies have been reported. For example, only two RCTs reported results pertaining to 6-month tumor recurrence rate and urethral stricture and the number of studies about non-laser ERBT was small. Such a limited sample size increases the likelihood of bias and reduces the statistical power of the analysis, making it difficult to draw reliable conclusions about these outcomes. Further researches in these areas should be encouraged. Last, lack of long-term follow-up data. Long-term follow-up studies are needed, this could involve extending the follow-up period of existing cohorts or initiating new studies with a focus on long-term outcomes.

## Conclusion

Our results show that ERBT is safer and more effective for NMIBC. ERBT can improve the rate of identification of detrusor muscle in specimens and reduce the 3-month, 6-month tumor recurrence rate and recurrence rate of the same site 1 year after surgery. In addition, ERBT has fewer complications and shorter catheter indentation time and hospital stay. The laser ERBT can also decrease tumor residual rate and re-TURBT. Nevertheless, the considerable heterogeneity observed in some of these outcomes cannot be overlooked, and the promising findings should be interpreted with greater caution. And further well-designed RCTs are needed to ascertain these results.

## Data Availability

The datasets are available from the corresponding author on reasonable request.

## Research registration unique identifying number (UIN)

The review was registered with the International Prospective Register of systematic reviews (PROSPERO) (CRD42024588191).
